# The substance use profiles of adults who sought mental health and addiction services through a centralized intake process in Nova Scotia (2020–2021)

**DOI:** 10.3389/fpsyt.2024.1476982

**Published:** 2024-12-13

**Authors:** Matiwos Soboka, Philip G. Tibbo, Sherry H. Stewart, Patryk Simon, JianLi Wang

**Affiliations:** ^1^ Department of Community Health and Epidemiology, Faculty of Medicine, Dalhousie University, Halifax, NS, Canada; ^2^ Department of Psychiatry, Faculty of Medicine, Dalhousie University, Halifax, NS, Canada; ^3^ Department of Psychology and Neuroscience, Faculty of Science, Dalhousie University, Halifax, NS, Canada; ^4^ Mental Health and Addiction Program, Nova Scotia Health, Halifax, NS, Canada

**Keywords:** substance use, substance use disorder, mental health problems, Canada, polysubstance use

## Abstract

**Background:**

Despite the increasing substance use in Canada, our understanding of how substance use varies based on the intersections of gender, ethnicity/race, and income sources among preclinical populations remains limited. Thus, this study aimed to investigate, among clients of mental health and addiction (MHA) intake in Nova Scotia: 1) the prevalence of substance use by gender, ethnicity, and income source; 2) the routes of substance administration; 3) factors associated with substance use. Understanding how gender, ethnicity, and income sources intersect to influence substance use patterns is essential for designing prevention and treatment strategies tailored to an individual’s unique needs. Additionally, exploring the various routes of substance administration can provide insight into potential health risks, helping to inform harm reduction strategies.

**Methods:**

This cross-sectional study included 22,500 adults who contacted MHA central intake in Nova Scotia in 2020 and 2021. Clients were assessed for substance use, substance use frequency, route of substance administration, and mental and physical health problems. The prevalence of substance use was examined as a function of gender, ethnicity, and income source. Multinomial logistic regression was used to investigate factors associated with substance use.

**Results:**

Among the included MHA Intake clients, 36.1% reported daily substance use. The highest prevalence of daily substance use was identified among homeless (69.7%) and non-White men on social assistance/disability (60.9%). Also, non-White individuals on social assistance/disability were more likely to engage in frequent (aOR = 2.66, 95% =1.64, 4.30) and daily (aOR = 2.82, 95% CI: 2.08, 3.82) substance use compared to White individuals. Being young (aged 19-29), lack of access to private insurance, current/past mental illness, moderate/high suicide risk, and presence of two or more psychosocial stressors, were associated with occasional, frequent, and daily substance use alike.

**Conclusions:**

The high prevalence of daily substance use among MHA Intake service users in Nova Scotia highlights the need for prevention and treatment strategies to address individual and structural level factors contributing to daily substance use.

## Introduction

Substance use is a major public health concern globally (1). Disability-adjusted life years (DALYs) are often used as a measure of impact of disease states or health behaviors on health-related quality of life. One DALY represents the loss of the equivalent of one year of full health. In 2016, 4.2% of DALYs globally were attributable to alcohol use, while 1.3% were attributable to other substance use (1). Annually, about 11.8 million deaths are linked to substance use (2), with alcohol alone causing three million deaths worldwide (3, 4). In North America, in 2019, substance use disorders (SUDs) ranked 5^th^ for years lived with disability (YLDs) and 15^th^ for years of life lost (YLLs) (5). Among countries in South and North America, Canada ranks second in terms of DALYs (5). Furthermore, Canada experiences approximately 67,000 deaths each year as a result of substance use (6).

Based on data from the 2012 Canadian Community Health Survey – Mental Health, about 6 million Canadians (21.6%) met the criteria for SUD in their lifetime (7). In Nova Scotia, the lifetime prevalence of SUD was 30.2%, the second highest in the Canadian provinces (8). SUD and mental illnesses often co-occur (9). Substance use can exacerbate symptoms of mental illnesses, while conversely, mental illnesses can drive individuals towards substance use as a form of coping or self-medication (10). A study conducted in Ontario showed that the prevalence of SUD varies from 17.1% among individuals with anxiety disorder to 34% among individuals with personality disorder (11). Moreover, polysubstance use is common among those with mental health disorders. For example, a study conducted in Nova Scotia showed that the prevalence of comorbid alcohol and cannabis use disorders among patients with a psychotic disorder was 50.0%, while the prevalences of alcohol use disorder alone and cannabis use disorder use alone were 12.5% and 20.8%, respectively (12). SUD among individuals with mental illnesses can lead to misdiagnosis, delayed intervention, relapse, poor prognosis, and poorer overall health (13). Thus, understanding the substance use profile among individuals with mental health needs is essential for tailoring effective interventions, addressing their specific needs, managing comorbidities, and improving treatment outcomes (14).

Most of the current studies about substance use are predominantly centered on clients already engaged in mental health and addiction services or on the general population. Less attention has been paid to individuals in the early stages of help-seeking. To the best of our knowledge, no study has investigated substance use profiles among the ‘pre-clinical’ population of those seeking mental health and addiction (MHA) services but who have yet to see a clinician. Also, no study has examined substance use disparities based on gender, race, or ethnicity, and socio-economic status among this population. Understanding how gender, ethnicity, and income sources intersect to influence substance use patterns among individuals seeking MHA services is essential for designing prevention and treatment strategies tailored to an individual’s unique needs. Additionally, understanding the epidemiology of substance use and its intersectionality with socio-demographic features in this population has significant implications for planning MHA services. Furthermore, exploring substance use profiles among individuals with mental health needs is pivotal for developing early interventions, personalized treatment plans, and targeted resource allocation. Examining the various routes of substance administration among individuals seeking MHA services can provide insight into potential health risks, helping to inform harm reduction strategies. Therefore, the objectives of this study were to investigate, among MHA intake clients in Nova Scotia: 1) the prevalence of substance use by gender, ethnicity, and income source; 2) the routes of substance administration; and (3) factors associated with substance use.

## Materials and methods

### Participants

All clients aged 19-64 years who were assessed by MHA Intake between January 2020 and December 2021 were included. The MHA Central Intake was established in 2019 by the Department of Health and Wellness of Nova Scotia as the entry point of MHA specialty services in the province and is the primary single entry-point of MHA services in Nova Scotia for this age range (individuals 18 years and younger are service through the child and adolescent system, and those 65 years and older are referred directly to geriatric specialty services). Individuals with symptoms of mental health and/or addiction problems living in any region of Nova Scotia (Northern, Eastern, Western, and Central zones) can directly contact MHA central intake using a toll-free telephone number. This central intake screens individuals with mental health and/or addiction problems and promptly links them to the appropriate level of mental health and addiction care. The intake process involves a semi-structured interview with the client by a clinician (e.g., clinical therapist, social worker, or registered nurse) over the telephone or in person. The information gathered during the interview was recorded on the electronic Intake Assessment form, which, once finalized, becomes an integral part of the individual’s permanent health record (15). This study was a secondary data analysis using existing de-identified data. Given the large number of clients in the database and vast area where they lived, obtaining informed consent from each client was not feasible. This study was approved by the Research Ethics Board of the Nova Scotia Health Authority. Individuals at higher risk of suicide were referred to psychiatrist for further evaluation, while those at mild and moderate risk received support from health professionals and psychologists working in the MHA Central Intake.

### Measures

#### Substance use measurements

The current substance use screening by MHA Intake included current use of alcohol, cannabis, hallucinogens, inhalants, opioids, sedatives/hypnotics, stimulants, and tobacco. The frequency of using these substances was evaluated via a questionnaire and included options of 2-4 times a month, 2-3 times a week, four or more times a week, and daily. The method(s) of administering each substance used was also queried, including oral, intravenous, inhaling, intramuscular, subcutaneous, smoking, snorting, transdermal patch, and/or rectal administration routes. The frequency of substance use was recoded into three categories of use: occasional use (2-4 times a month), frequent use (2-3 times a week, and four or more times a week), and daily use.

#### Presence of current or past provisional diagnosis of mental disorders

The following mental health problems were assessed and current/provisional diagnoses were made based on the client’s report: depression, anxiety disorder, bipolar disorder, attention-deficit/hyperactivity disorder, adjustment disorder, autism, eating disorder, neurocognitive disorder, obsessive-compulsive disorder, personality disorder, psychotic disorder, posttraumatic stress disorder, and substance use disorder. We aggregated the presence of current or past provisional diagnoses of mental health disorders into a single variable with three levels: no mental health disorder (coded as 0); provisional diagnosis of one current/past mental illness (coded as 1); or two or more provisional diagnoses of current/past mental illnesses (coded as 2).

#### Presence of current or history of medical problems

Clients were interviewed for the presence current/past medical illnesses and we aggregated the presence of current or past provisional diagnoses of medical illnesses into a single variable with three levels: no current/past medical illness (coded as 0); a provisional diagnosis of one current/past medical illness (coded as 1); or two or more provisional diagnoses of current/past medical illnesses (coded as 2).

#### Suicide risk

Clients were assessed for past suicide attempts, suicidal ideation in the two weeks before the interview, and current suicidal ideation (at the time of the interview). The clinician who conducted the interview classified clients into low, moderate, or high suicide risk levels based on a suicide risk assessment and intervention tool (15).

#### Psychosocial stressors

Clients were assessed to determine if they had experienced current/past psychosocial stressors in the following areas: childhood adversity, abuse or other trauma, economic/financial, educational/school, ethnic/cultural, spiritual/religious beliefs, family and/or significant relationship, social relationships, housing or legal issues, leisure/recreational, military, parent/guardian–child conflict, or physical health/disability, and how these stressors affected their functioning (15). In this analysis, we classified psychosocial stressors into three categories: the absence of any such stressors (coded as 0); the experience of one such stressor (coded as 1); or the experience of two or more psychosocial stressors (coded as 2).

#### Demographic and socio-economic information

Clients were queried on gender identity, age, marital status, income source(s), ethnicity, living conditions, access to employee assistance programs (EAP) or private insurance, and Nova Scotia health zone (Northern, Eastern, Western, or Central).

### Data imputation

We first examined the frequency of each variable, its distribution, and rates of missing values. We selected 128 variables with missing values for imputation based on our objectives. Multiple Imputation by Chained Equations (MICE) was used to impute variables with missing values (missing at random). We opted for MICE as our method of choice because of its flexibility in generating multiple predictions for each missing value. This approach relies on the variable’s distribution, the observed values for a given participant, and the correlations observed in the dataset for other participants (16, 17). In this study, the imputed variables with missing values ranged from 0.004% (for bromazepam [a sedative/hypnotic] route of administration) to 20.9% (impact of mood symptoms on functioning). Traditionally, a small number of imputations (five to ten) are commonly used (18, 19). However, to achieve a better estimate of standard error, a higher number of imputations are recommended, which is at least equal to the average percentage rate of missing values, as a rule of thumb (18, 19). Considering the average percentage rate of missing values in our study (i.e., 0.76%), we used five imputations with a maximum iteration of 20. The imputed datasets were used to complete variables with missing values and Rubin’s rules were used to pool estimates in our analysis (20).

### Data analysis

Descriptive statistics were used to report on socio-demographic characteristics of the sample and rates of use of each substance. To reduce the complexity of the analysis and increase the interpretability of the results, for objective one, the frequency of using substances such as alcohol, opioids, stimulants, cannabis, hallucinogens, sedatives/hypnotics, tobacco, and other substances (nitrous oxide, cough syrup, caffeine pills) was aggregated to yield one composite variable labelled “frequency of substance use.” To derive this composite frequency of substance use variable, we retained the highest frequency score from among the individual frequency of alcohol, opioid, amphetamine/methamphetamine, cocaine, cannabis, hallucinogens, sedatives, and/or other substance use items. For example, if the client’s responses were ‘not using’ for alcohol, ‘2-4 times a month’ for opioids, ‘2-3 times a week’ for cocaine and amphetamine/methamphetamine, and ‘daily’ for cannabis, their overall frequency of substance use was coded as ‘daily’. Then, the client’s overall frequency of substance use was re-coded into three categories: occasional use (2-4 times a month), frequent use (2-3 times a week and four or more times a week), or daily use.

We then calculated the proportion of the sample who were using substances and the proportions using at each frequency category. We also computed these two substance indices as a function of gender, ethnicity/race, and income source. For objective two, we calculated the proportion each route of administration for users of each substance. Multinomial logistic regression was employed to investigate factors associated with occasional, frequent, and daily substance use compared to abstaining from substance use. First, we included demographic and socio-economic variables as predictors in the multinomial logistic regression model without introducing any interactions between variables. Then, two-way interactions between gender and other predictor variables were included in the multinomial logistic regression model, along with demographic and socio-economic variables, history of mental and physical illnesses, suicide risk, and psychosocial stressors. Pooled adjusted odds ratios and corresponding 95% confidence intervals were used to estimate the strength of association. The analysis was conducted utilizing R software (version 4.2.3).

## Results

**Figure 1:**
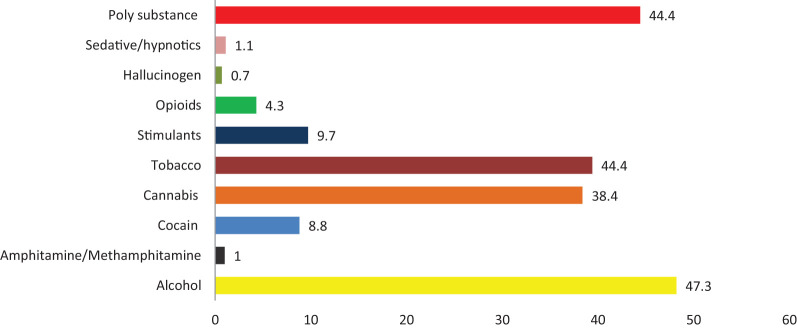
The proportion of substance use among clients aged 19-64 who were assessed by the Nova Scotia Mental Health and Addiction intake in 2020 and 2021.

A total of 22,500 clients who contacted MHA intake from 2020 to 2021 were included in this study (6451 men, 8798 women, 186 non-binary, and 7065 who did not specify their gender). The most frequently reported substances used were alcohol (47.3%), tobacco (44.4%), cannabis (38.4%), and cocaine (8.8%) (see **Figure 1**). The prevalence of polysubstance use was 44.4%.

### Frequency of substance use

Among the participants, 36.1% used a substance daily, while 10.0% and 12.4% used it frequently and occasionally, respectively. A significantly higher prevalence of daily substance use was observed among men (44.7%, p < 0.001) than among women (29.3%), non-binary individuals (32.3%), and those who did not specify their gender (36.7%). Among homeless participants, 69.7% reported daily substance use, which was about two times higher than the prevalence observed among individuals living in private homes, apartments, or rented rooms (35.3%) (see **Table 1**).

**Table 1:** Socio-demographic characteristics and observed prevalence of substance use and of three categories of substance use frequency (occasional, frequent, or daily).

Variables	N	Substance use N (%)	Occasional users N (%)	Frequent users N (%)	Daily users N (%)
Gender Identity					
Woman	8798	6235 (70.87)	1127 (12.81)	892 (10.14)	2581 (29.34)
Man	6451	5286 (81.94)	702 (10.88)	741 (11.49)	2885 (44.72)
Non-binary	186	140 (75.27)	25 (13.44)	24 (12.9)	60 (32.26)
Did not specify gender identity	7065	5692 (80.57)	946 (13.39)	600 (8.49)	2595 (36.73)
Age groups					
19-29 years old	7996	6286 (78.61)	1107 (13.84)	857 (10.72)	3007 (37.61)
30-49 years old	9735	7668 (78.77)	1166 (11.98)	971 (9.97)	3723 (38.24)
50-64 years old	4769	3398 (71.27)	527 (11.05)	429 (9.00)	1391 (29.17)
Marital status					
Married/common-law/partnership	11421	8501 (74. 43)	1503 (13.16)	1126 (9.86)	3664 (32.08)
Single	7815	6338 (81.01)	916 (11.72)	798 (10.21)	3343 (42.78)
Separated/Divorced/ Widowed	3264	2513 (76.99)	381 (11.67)	333 (10.20)	1114 (34.13)
Income source					
Employment	9947	7594 (76.34)	1402 (14.09)	1161 (11.67)	3155 (31.72)
Spouse/partner	648	422 (65.12)	86 (13.27)	49 (7.56)	162 (25.00)
Employment Insurance/pension	3831	3047(79.54)	428 (11.17)	372 (9.71)	1574 (41.09)
Social assistance/disability	4454	3472 (77.95)	450 (10.10)	348 (7.81)	1841 (41.33)
Other	1557	1222 (78.48)	222 (14.26)	152 (9.76)	545 (35.00)
None	2063	1595 (77.31)	212 (10.28)	175 (8.48)	844 (40.91)
Living status					
Private home/apartment/rented room	21409	16453 (76.85)	2734 (12.77)	2193 (10.24)	7550 (35.27)
Board and care	352	264 (75.00)	22 (6.55)	29 (8.24)	154 (43.75)
Assisted living/group home	110	51 (46.36)	8 (7.27)	1 (0.91)	15 (13.64)
Homeless	479	456 (95.20)	25 (5.22)	32 (6.68)	334 (69.73)
Others	150	128 (85.33)	14 (9.33)	8 (5.33)	65 (43.33)
Health zone					
Central	10497	8388 (79.91)	1410 (13.43)	1274 (12.14)	3933 (37.47)
Eastern	4524	3128 (69.14)	438 (9.68)	286 (6.32)	1602 (35.41)
Northern	4080	3175 (77.82)	406 (9.95)	322 (7.89)	1605 (39.34)
Western	3399	2661 (78.29)	546 (16.06)	375 (11.03)	981 (28.86)
Race and Ethnicity					
White	13559	10578 (78.01)	1684 (12.42)	1506 (11.11)	5081 (37.47)
First Nation	864	770 (89.12)	100 (11.57)	59 (6.83)	483 (55.9)
Black	360	291 (80.83)	48 (13.33)	44 (12.22)	140 (38.89)
Asian	195	103 (52.82)	32 (16.41)	14 (7.18)	30 (15.38)
Other	454	335 (73.79)	51 (11.23)	35 (7.71)	163 (35.9)
Declined to answer race/ethnicity	7068	5275 (74.63)	885 (12.52)	599 (8.47)	2224 (31.47)

Other income: Child Tax Benefit, Worker’s Compensation Board, Savings, Canada Emergency Response Benefit, Parents, family, Student loans, spouse, none

Other living condition: Group home, transition house, jail, halfway house, hostel, and hotel

High prevalences of daily substance use were observed among non-White men whose income source was social assistance or disability support (60.9%) or employment insurance/pension (56.4%) (see **Table 2**).

**Table 2:** Prevalence of substance use frequency at the intersection of gender, ethnicity/race, and income sources.

Gender	Ethnicity	Income sources	Stratum	Non-user N(%)	Occasional substance use N(%)	Frequent substance use N(%)	Daily substance use N(%)
Man	White	Employment	1872	559 (29.86)	242 (12.93)	282 (15.06)	789 (42.15)
		Employment insurance/pension	746	196 (26.27)	61 (8.18)	95 (12.73)	394 (52.82)
		social assistance/disability	741	234 (31.58)	67 (9.04)	67 (9.04)	373 (50.34)
		Spouse/Others	258	87 (33.72)	30 (11.63)	18 (6.98)	123 (47.67)
		None	402	127 (31.59)	46 (11.44)	41 (10.20)	188 (46.77)
	Non-White	Employment	218	74 (33.94)	36 (16.51)	21 (9.63)	87 (39.91)
		Employment insurance/pension	101	26 (25.74)	10 (9.90)	8 (7.92)	57 (56.44)
		social assistance/disability	174	41 (23.56)	10 (5.75)	17 (9.77)	106 (60.92)
	Declined to answer	Employment	798	320 (40.10)	87 (10.90)	98 (12.28)	293 (36.72)
		Employment insurance/pension	363	132 (36.36)	42 (11.57)	38 (10.47)	151 (41.60)
		social assistance/disability	368	152 (41.30)	37 (10.05)	24 (6.52)	155 (42.12)
		Spouse/Others	100	48 (48.00)	9 (9.00)	6 (6.00)	37 (37.00)
		None	214	103 (48.13)	15 (7.01)	17 (7.94)	79 (36.92)
Women	White	Employment	2543	1163 (45.73)	374 (14.71)	301 (11.84)	705 (27.72)
		Employment insurance/pension	809	372 (45.98)	82 (10.14)	85 (10.51)	270 (33.37)
		social assistance/disability	1037	526 (50.72)	96 (9.26)	91 (8.78)	324 (31.24)
		Spouse/Others	596	267 (44.80)	103 (17.28)	67 (11.24)	159 (26.68)
		None	426	177 (41.55)	42 (9.86)	44 (10.33)	163 (38.26)
	Non-White	Employment	266	126 (47.37)	38 (14.29)	26 (9.77)	76 (28.57)
		social assistance/disability	178	58 (32.58)	9 (5.06)	17 (9.55)	94 (52.81)
		Spouse/Others	81	45 (55.56)	7 (8.64)	9 (11.11)	20 (24.69)
	Declined to answer	Employment	1206	631 (52.32)	185 (15.34)	118 (9.78)	272 (22.55)
		Employment insurance/pension	452	235 (51.99)	48 (10.62)	41 (9.07)	128 (28.32)
		social assistance/disability	518	255 (49.23)	54 (10.42)	40 (7.72)	169 (32.63)
		Spouse/Others	307	165 (53.75)	41 (13.36)	31 (10.10)	70 (22.80)
		None	214	108 (50.47)	27 (12.62)	14 (6.54)	65 (30.37)
Non-binary/Did not specify	White	Employment	1722	701 (40.71)	247 (14.34)	202 (11.73)	572 (33.22)
		Employment insurance/pension	716	236 (32.96)	80 (11.17)	66 (9.22)	334 (46.65)
		social assistance/disability	801	298 (37.20)	108 (13.48)	67 (8.36)	328 (40.95)
		Spouse/Others	507	216 (42.60)	63 (12.43)	50 (9.86)	178 (35.11)
		None	383	129 (33.68)	43 (11.23)	30 (7.83)	181 (47.26)
	Non-White	Employment	208	89 (42.79)	46 (22.12)	15 (7.21)	58 (27.88)
		social assistance/disability	167	42 (25.15)	18 (10.78)	8 (4.79)	99 (59.28)
		Spouse/Others	71	28 (39.44)	10 (14.08)	6 (8.45)	27 (38.03)
	Declined to answer	Employment	1114	566 (50.81)	147 (13.20)	98 (8.80)	303 (27.20)
		Employment insurance/pension	473	205 (43.34)	80 (16.91)	30 (6.34)	158 (33.40)
		social assistance/disability	470	209 (44.47)	51 (10.85)	17 (3.62)	193 (41.06)
		Spouse/Others	245	122 (49.80)	40 (16.33)	11 (4.49)	72 (29.39)
		None	226	109 (48.23)	22 (9.73)	16 (7.08)	79 (34.96)

Among clients who used amphetamine/methamphetamine, cannabis, and opioids, 52.4%, 60.1%, and 69.0% reported daily use, respectively (see **Table 3**).

**Table 3:** Frequency of specific types of substance use among clients aged 19-64 who were using substances (N=22,500). .

Type of substances	Frequency of using substance, N (%)			
	2-4 times a month	2-3 times a week	Four or more times weekly	Daily
Alcohol	2712 (25.49)	1189 (11.18)	664 (6.34)	2707 (25.44)
Opioid	52 (5.63)	61 (6.61)	69 (7.48)	637 (69.01)
Amphetamine/ methamphetamine	33 (14.16)	27 (11.59)	17 (7.30)	122 (52.36)
Cocaine	381 (19.26)	287 (14.51)	207 (10.47)	842 (42.57)
Cannabis	2006 (23.25)	829 (9.61)	415 (4.81)	5187 (60.12)
Hallucinogen	73 (47.10)	16 (10.32)	23 (14.84)	4 (2.58)
Sedatives/hypnotics	28 (11.38)	51 (20.73)	28 (11.38)	125 (50.81)

### Route of substance administration

Smoking was a common route of administration among participants using cannabis (80.0%), cocaine (38.3%), and amphetamine/methamphetamine (28.3%), whereas injection was a common route of administration among participants using opioids (52.2%) (**Supplementary Table 1**).

### Factors associated with frequency of substance use

Multinomial logistic regression modelling revealed that men were more likely to engage in occasional (aOR =1.48, 95% CI: 1.24, 1.76), frequent (aOR =2.12, 95% CI: 1.77, 2.54), and daily substance use (aOR = 2.60, 95% CI: 2.27, 2.97) than women. Also, non-binary individuals or those not specifying their gender had higher odds of occasional (aOR = 1.19, 95% CI: 1.00, 1.41), frequent (aOR =1.23, 95% CI: 1.02, 1.48), and daily (aOR =1.39, 95% CI: 1.21, 1.58) substance use compared to women. In comparison to individuals residing in a private home, apartment, or rented home, individuals experiencing homelessness or residing in other living conditions had increased odds of daily substance use (aOR = 1.93, 95%CI = 1.57, 2.37). Non-White individuals, as compared to those of White ethnicity/race, had higher odds of daily substance use when their income source was from social assistance or disability (aOR = 2.82, 95% CI: 2.08, 3.82), or employment insurance or pension (aOR = 1.68, 95% CI: 1.16, 2.42).

The presence of two or more mental illnesses currently or in the past was associated with increased odds of occasional, frequent, and daily substance use compared to no mental health conditions. In comparison to the absence of psychosocial stressors, experiencing two or more psychosocial stressors was associated with higher odds of engaging in occasional, frequent, and daily substance use (see **Table 4**).

**Table 4:** Multinomial logistic regression: Factors associated with frequency of substance use.

Variables	Frequency of substance use		
	Occasional use aOR (95%CI, lower, upper)	Frequent use aOR (95%CI, lower, upper)	Daily use aOR (95%CI, lower, upper)
Gender			
Women			
Man	1.48 (1.24, 1.76)	2.12 (1.77, 2.54)	2.60 (2.27, 2.97)
Non-binary/did not specify	1.19 (1.00, 1.41)	1.23 (1.02, 1.48)	1.39 (1.21, 1.58)
Age groups			
19-29 years old	1.65 (1.44, 1.89)	1.48 (1.28, 1.72)	1.72 (1.55, 1.90)
30-49 years old	1.38 (1.22, 1.56)	1.36 (1.19, 1.56)	1.71 (1.56, 1.87)
50-64 years old			
Race/Ethnicity			
White			
Non-White	0.98 (0.72, 1.34)	0.67 (0.46, 0.98)	0.92 (0.72, 1.17)
Declined to answer	1.00 (0.83, 1.21)	1.01 (0.81, 1.24)	0.98 (0.84, 1.13)
Marital status			
Married/common-law/partnership			
Single	1.02 (0.93, 1.13)	1.25 (1.12, 1.40)	1.34 (1.24, 1.44)
Separated/Divorced/widowed	1.02 (0.89, 1.16)	1.19 (1.03, 1.37)	1.06 (0.96, 1.17)
Income sources			
Employment			
Employment Insurance/pension	0.67 (0.53, 0.84)	0.91 (0.71, 1.16)	1.18 (1.00, 1.39)
Social assistance/disability	0.60 (0.48, 0.75)	0.71 (0.57, 0.90)	0.88 (0.75, 1.03)
Spouse/partner/other	0.97 (0.77, 1.23)	0.90 (0.69, 1.18)	0.83 (0.68, 1.01)
None	0.77 (0.57, 1.04)	0.83 (0.60, 1.16)	1.22 (0.99, 1.50)
Living status			
Private home/apartment/rented room			
Board and care/Assisted living/group home	0.44 (0.30, 0.65)	0.55 (0.37, 0.82)	0.66 (0.53, 0.82)
Homeless/others	0.75 (0.52, 1.08)	0.93 (0.64, 1.33)	1.93 (1.57, 2.37)
Access to EPA or private insurance			
No	1.09 (1.00, 1.20)	1.10 (1.00, 1.21)	1.47 (1.38, 1.58)
Yes			
Health Zones			
Western			
Eastern	0.64 (0.56, 0.73)	0.47 (0.40, 0.56)	0.83 (0.75, 0.91)
Northern	0.73 (0.64, 0.83)	0.63 (0.55, 0.73)	1.00 (0.91, 1.09)
Western	1.22 (1.06, 1.39)	0.93 (0.80, 1.09)	0.85 (0.76, 0.94)
Suicide risk			
No			
Mild	1.56 (1.41, 1.71)	1.52 (1.37, 1.69)	1.71 (1.59, 1.83)
Moderate/High	1.40 (1.12, 1.76)	1.34 (1.04, 1.73)	1.58 (1.34, 1.86)
Current/past mental health problems			
0			
1	1.34 (1.19, 1.51)	1.28 (1.13, 1.46)	1.57 (1.44, 1.71)
2+	1.34 (1.21, 1.49)	1.21 (1.08, 1.36)	1.43 (1.33, 1.55)
Current/past medical health problems			
0			
1	1.09 (0.97, 1.23)	0.97 (0.85, 1.11)	1.25 (1.15, 1.36)
2+	1.08 (0.91, 1.28)	0.91 (0.75, 1.10)	1.03 (0.91, 1.17)
Psychosocial stressors			
0			
1	1.58 (1.39, 1.80)	2.22 (1.92, 2.55)	1.96 (1.79, 2.15)
2+	1.96 (1.76, 2.19)	2.52 (2.22, 2.85)	2.32 (2.14, 2.51)
Two-way interaction of gender, ethnicity and income sources			
Gender*Ethnicity/Race			
Woman			
Man*Non-White	1.21 (0.80, 1.83)	1.04 (0.66, 1.63)	0.92 (0.69, 1.22)
Man * Decline to answer	0.77 (0.60, 0.99)	0.81 (0.63, 1.06)	0.81 (0.68, 0.96)
Non-binary/Did not specify *Non-White	1.39 (0.95, 2.05)	0.80 (0.50, 1.29)	0.88 (0.66, 1.17)
Non-binary* Did not specify/declined to answer	0.82 (0.66, 1.02)	0.69 (0.53, 0.89)	0.86 (0.73, 1.01)
Gender*Income sources			
Employment			
Man*Employment Insurance/pension	1.21 (0.88, 1.67)	1.14 (0.83, 1.57)	1.11 (0.89, 1.39)
Man * Social assistance/disability	1.25 (0.91, 1.70)	0.85 (0.62, 1.18)	0.99 (0.80, 1.22)
Man * Spouse/partner/Other	0.75 (0.49, 1.15)	0.43 (0.26, 0.70)	1.00 (0.74, 1.36)
Man * None	0.98 (0.65, 1.47)	0.75 (0.49, 1.15)	0.66 (0.50, 0.87)
Non-binary/did not specify * Employment Insurance/pension	1.46 (1.09, 1.96)	1.00 (0.72, 1.40)	1.25 (1.00, 1.55)
Non-binary/did not specify * Social assistance/disability	1.60 (1.21, 2.12)	0.92 (0.66, 1.28)	1.24 (1.01, 1.52)
Non-binary/did not specify * Spouse/partner/other	0.92 (0.67, 1.27)	0.78 (0.54, 1.14)	1.06 (0.82, 1.37)
Non-binary/did not specify * None	1.13 (0.76, 1.68)	0.93 (0.59, 1.45)	1.00 (0.76, 1.32)
Race/Ethnicity*Income sources			
White			
Non-White * Employment Insurance/pension	1.43 (0.87, 2.35)	1.17 (0.63, 2.20)	1.68 (1.16, 2.42)
Non-White * Social assistance/disability	0.98 (0.62, 1.56)	2.66 (1.64, 4.30)	2.82 (2.08, 3.82)
Non-White * Spouse/partner/Other	0.61 (0.35, 1.06)	1.30 (0.70, 2.45)	1.07 (0.70, 1.61)
Non-White * None	0.64 (0.35, 1.19)	1.01 (0.50, 2.02)	1.07 (0.72, 1.60)
Decline * Employment insurance/pension	1.42 (1.08, 1.86)	1.03 (0.77, 1.39)	0.90 (0.74, 1.10)
Decline * Social assistance/disability	1.17 (0.89, 1.53)	0.99 (0.72, 1.35)	1.29 (1.07, 1.55)
Decline * Spouse/partner/Other	0.98 (0.71, 1.36)	0.92 (0.62, 1.37)	0.98 (0.76, 1.27)
Decline * None	0.83 (0.58, 1.20)	0.82 (0.54, 1.22)	0.80 (0.62, 1.02)

## Discussion

Our study revealed large proportions of the MHA Intake clients reported daily substance use. A particularly high prevalence of daily substance use was observed among non-White men with income sources from social assistance or disability support. Being non-White with income sources from social assistance or disability and employment insurance or pension, homelessness/others, and the presence of two or more mental or medical illnesses were associated with higher odds of daily substance use.

In this study, about one-third (36.1%) of our sample of individuals seeking mental health and addictions services reported daily substance use. More specifically, the observed prevalence of daily opioid (69.0%) and cannabis (60.1%) use in our study was higher than the prevalence of daily opioid (40%) and cannabis (36%) use reported in a study conducted in Vancouver (21). The observed differences may be due to variations in the study populations. Our study population consisted of individuals with mental illnesses and addiction, while the Vancouver study focused on individuals who use drugs and experienced chronic pain. Additionally, the Vancouver study had a smaller sample size (1,476 participants) compared to our study, which may contribute to the observed differences. In our study, a large proportion of daily amphetamine/methamphetamine (52.4%), sedative/hypnotics (50.8%), and cocaine (42.6%) use was reported. The high prevalence of daily opioids use among clients of MHA Intake may lead to opioid use disorder and exposes these individuals to overdose risk (22).

The high prevalence of daily substance use in our study can be attributed to the unique nature of our study population: individuals in the early stage of seeking mental health and addiction treatment services. These individuals may use substances daily as a form of self-medication for symptoms of mental health problems (23). Additionally, the high prevalence of substance use, particularly daily substance use, observed in our study has important clinical implications since using substances can either exacerbate the existing mental health problems or lead to the development of new conditions (e.g., addiction, physical health problems) and drop out once they are engaged in services (24). Furthermore, the high prevalence of daily substance use in our study implies the importance of an integrated care model that addresses both mental health problems and substance use simultaneously, as well as targeted prevention and intervention strategies aimed at reducing substance use among vulnerable individuals. Also, this finding indicates the need for a broader and more nuanced approach to understanding how substance use interacts with mental health problems and psychiatric medications.

The high prevalence of polysubstance use observed in our study (44.4%) has significant clinical implications. Polysubstance use not only exacerbates symptoms of mental health problems but can also interfere with the efficacy of psychiatric medications (25, 26). Additionally, the concurrent use of various substances can mask underlying mental health problems and complicate their treatment (27). Moreover, polysubstance use can increase the risk of overdose, cognitive dysfunctions, and aggressiveness including violent criminal behavior (25). Using various substances, particularly when novel psychoactive substances are used for adulteration, can lead to in unpredictable health consequences and complicated treatment and harm reduction efforts (25, 28).

Our study found a significant variation in substance use across socio-demographic characteristics. In line with previous studies (29, 30), we found a high prevalence of daily substance use among men (44.7%) compared to women (29.3%) and non-binary/gender non-specified individuals (36.7%). This gender difference can be at least partially attributed to sociocultural factors, including societal norms, expectations, and culturally-sanctioned gender roles (30). Though the prevalence of daily substance use among women was lower than among men, women are at higher risk of experiencing acute and long term consequences of substance use than men (30), making the relatively high rates of daily use seen among women in our sample (29.3%) of clinical concern. Among homeless individuals, about two-thirds (69.7%) were engaged in daily substance use. This could be due to the fact that substance use disorder can lead to job loss, disruption of social ties, and loss of housing, which results in homelessness (31). In Canada, for instance, about 25% of Canadians reported that substance use was responsible for their most recent housing loss (32). On the other hand, homelessness-related stress may also lead to substance use to cope (33).

An individual’s socio-economic condition significantly influences their substance use and the development SUD. Poverty not only increases substance use but also exacerbates the risks associated with SUD (34). In line with studies conducted in the USA (35–37), we found a very high prevalence of daily substance use among individuals with income sources from social assistance or disability support (41.3%) and employment insurance or pension (41.1%). This could be due to individuals with economic problems resorting to substance use to cope with difficult life situations and stress related to financial hardships (38). Additionally, individuals with insecure sources of income may face challenges in accessing mental and addiction treatment services, and as a result, substances may be used as self-medication (36). We also found that the majority of non-White men with income sources from social assistance/disability (60.9%) and employment insurance/pension (56.4%) engaged in daily substance use. Since non-White races/ethnicities were disproportionately using substances, developing targeted interventions and promoting equitable access to treatment and support services are crucial.

In this study, the presence of two or more mental health problems was associated with increased odds of daily substance use. This could be due to the fact that individuals with mental health problems may turn to substance use as a self-medication to temporarily alleviate symptoms of mental illnesses (39). Additionally, individuals with mental health problems may use substances to cope with stress, as a source of pleasure, and for socialization purposes (39, 40). Conversely, in the longer term, substance use affects the brain’s neurobiology and leads to changes in mood, cognition, and behavior, which contribute to the development of mental illnesses or exacerbation of symptoms (39). We also found that having two or more psychosocial stressors was associated with all levels of substance use: occasional, frequent, and daily. This may stem from the tendency of individuals facing psychosocial stressors to utilize substances as a coping mechanism (41). Over time, these stressors can increase the risk of initiating substance use and developing addiction (42).

This study is the first provincial-level analysis providing evidence regarding substance use disparities, considering the intersection of gender, ethnicity, and income sources among clients seeking MHA services. This type of study is instrumental in identifying and developing plans to address health equity concerns and instituting intervention strategies that consider the unique needs of various subgroups in society. Also, what makes our study the first in Canada is the unique nature of our study population: individuals seeking MHA services with symptoms of unconfirmed mental health problems and addiction. However, our study has also some limitations. We used a cross-sectional study design that cannot establish a temporal relationship, making it difficult to know if, for example, social disability and mental illnesses precede and/or follow substance use. Moreover, due to social desirability bias, clients may not disclose detailed information about illegal drug use or even deny using it. Additionally, this study may not generalizable to all individuals with mental health problems and addiction across Canada. Moreover, we did not gather data regarding tobacco and other substance use frequency. Additionally, although the prevalence of opioid use was high, we did not gather data regarding opioid overdose and related emergency department visit or hospitalization. Also, we did not use standard tools or DSM-5 criteria to assess mental health problems.

## Conclusions

The prevalence of daily substance use was high in our sample of individuals seeking mental health and addictions services and varied by participant socio-demographic characteristics of gender identity, ethnicity/race, and/or income source. The highest prevalence of daily substance use was observed among non-White men whose income source was from social assistance or disability support and employment insurance/pension, indicating that prevention and treatment approaches should address these individual and structural level factors contributing to daily substance use. Being homeless/other living conditions (Group home, transition house, jail, halfway house, hostel, and hotel), having two or more medical or mental illnesses (current or past), and experiencing two or more psychosocial stressors were associated with daily substance use; further studies are needed to understand the temporal relationship between these variables and daily substance use.

## Data Availability

The findings of this study rely on data owned by Nova Scotia Health (NSH) Authority; thus, access may be granted, subject to approval from the data custodian. Requests to access the datasets should be directed to patryk.simon@nshealth.ca.
