# Supplementary material for: The substance use profiles of adults who sought mental health and addiction services through a centralized intake process in Nova Scotia (2020–2021)
Source: Front Psychiatry. 2024 Dec 13;15:1476982. doi: 10.3389/fpsyt.2024.1476982 (PMC11697425; doi:10.3389/fpsyt.2024.1476982)
Supplement: Supplementary file 1 [file Table1.docx]

**Supplementary Table 1: Route of administration of specific substances among patients aged 19-64 who were using substances (N=22,500).**

| Type of substances | Route of administration, N(%) | | | | | |
| --- | --- | --- | --- | --- | --- | --- |
|  | Injection (IV) | Oral | Snorting | Inhaling | Smoking | Rectal |
| Alcohol | - | 10639 (100.00) | - | - | - | - |
| Opioid | 482 (52.22) | 245 (26.54) | 160 (17.33) | 3 (0.33) | 12 (1.30) | 1 (0.11) |
| Amphetamine/  methamphetamine | 53 (22.75) | 37 (15.88) | 31 (13.30) | 5 (2.15) | 66 (28.33) | - |
| Cocaine | 360 (18.20) | 3 (0.15) | 809 (40.90) | 44 (2.22) | 757 (38.27) | - |
| Cannabis | - | 1321 (15.31) | 5 (0.06) | - | 6901 (79.98) | - |
| Hallucinogen | - | 133 (85.81) | 10 (6.45) | 1 (0.65) | 2 (1.29) | - |
| Sedatives/  hypnotics | 7 (3.61) | 148 (76.29) | 11 (5.67) | - | 5 (2.58) | - |
